# Supplementary material for: Attitude and Acceptance towards COVID-19 Booster Doses among Literacy Advantaged Population in Pakistan: A Cross-Sectional Study
Source: Vaccines (Basel). 2023 Jul 14;11(7):1238. doi: 10.3390/vaccines11071238 (PMC10383426; doi:10.3390/vaccines11071238)
Supplement: Supplementary file 1 [file vaccines-11-01238-s001.zip › Statistical results of Ph.D Participants.pdf]

PHDs different results

Work_Status

		Frequency	Percent	Valid Percent	Cumulative Percent
Valid	Employed	29	54.7	54.7	54.7
	Retired	6	11.3	11.3	66.0
	Unemployed	18	34.0	34.0	100.0
	Total	53	100.0	100.0	

Interactive_Profession

		Frequency	Percent	Valid Percent	Cumulative Percent
Valid	No	6	11.3	20.7	20.7
	Yes	23	43.4	79.3	100.0
	Total	29	54.7	100.0	
Missing	System	24	45.3		
Total		53	100.0		

Profession

		Frequency	Percent	Valid Percent	Cumulative Percent
Valid	Health or Allied Healthcare	13	24.5	56.5	56.5
	Others	10	18.9	43.5	100.0
	Total	23	43.4	100.0	
Missing	System	30	56.6		
Total		53	100.0		

92.5% had vaccine

Receipt_COVID_Vaccine

		Frequency	Percent	Valid Percent	Cumulative Percent
Valid	Not Sure	1	1.9	1.9	1.9
	No	3	5.7	5.7	7.5

	Yes	49	92.5	92.5	100.0
	Total	53	100.0	100.0	

41.5% had all three administered

Dose_of_vaccine

		Frequency	Percent	Valid Percent	Cumulative Percent
Valid	All of them	21	39.6	42.9	42.9
	1st Dose	2	3.8	4.1	46.9
	2nd Dose/Booster	25	47.2	51.0	98.0
	3rd Dose/Booster	1	1.9	2.0	100.0
	Total	49	92.5	100.0	
Missing	System	4	7.5		
Total		53	100.0		

34% had already had covid infection

Previous_COVID_Infection

		Frequency	Percent	Valid Percent	Cumulative Percent
Valid	Not Sure	9	17.0	18.4	18.4
	No	22	41.5	44.9	63.3
	Yes	18	34.0	36.7	100.0
	Total	49	92.5	100.0	
Missing	System	4	7.5		
Total		53	100.0		

15.1% reported any side effect of 3rd dose of vaccine

Side_Effects_3rd_Dose

		Frequency	Percent	Valid Percent	Cumulative Percent
Valid	No	41	77.4	83.7	83.7
	Yes	8	15.1	16.3	100.0
	Total	49	92.5	100.0	
Missing	System	4	7.5		
Total		53	100.0		

9.4% responded that 3rd dose of vaccine resulted in most side effects

Dose_with_more_side_effects

		Frequency	Percent	Valid Percent	Cumulative Percent
Valid	1	1	1.9	12.5	12.5
	2	2	3.8	25.0	37.5
	3	5	9.4	62.5	100.0
	Total	8	15.1	100.0	
Missing	System	45	84.9		
Total		53	100.0		

30.2% had already had a jab. 18.9% are ready to have it

Booster_Dose_Acceptance

		Frequency	Percent	Valid Percent	Cumulative Percent
Valid	Already have taken it	16	30.2	32.7	32.7
	May be	6	11.3	12.2	44.9
	No	13	24.5	26.5	71.4
	Only if job requirement	4	7.5	8.2	79.6
	Yes	10	18.9	20.4	100.0
	Total	49	92.5	100.0	
Missing	System	4	7.5		
Total		53	100.0		

56.6% said that they want vaccine for their own good health

Reason_for_Taking_3rd_Booster

		Frequency	Percent	Valid Percent	Cumulative Percent
Valid	Job requirement	1	1.9	2.3	2.3
	Protection of colleagues	3	5.7	7.0	9.3
	Protection of family health	4	7.5	9.3	18.6
	Protection of my own health	30	56.6	69.8	88.4
	Travel requirement	5	9.4	11.6	100.0
	Total	43	81.1	100.0	
Missing	System	10	18.9		

Total	53	100.0		

Encouragement_to_Get_Vaccination

		Frequency	Percent	Valid Percent	Cumulative Percent
Valid	Free vaccination	23	43.4	46.9	46.9
	Only if compulsory requirement for work or travel	26	49.1	53.1	100.0
	Total	49	92.5	100.0	
Missing	System	4	7.5		
Total		53	100.0		

50.9% thinks that vaccine is beneficial

Thoughts_on_Benefit

		Frequency	Percent	Valid Percent	Cumulative Percent
Valid	Not Sure	19	35.8	38.8	38.8
	No	3	5.7	6.1	44.9
	Yes	27	50.9	55.1	100.0
	Total	49	92.5	100.0	
Missing	System	4	7.5		
Total		53	100.0		

67.9% says vaccine is beneficial

fficacy_of_Vaccine

		Frequency	Percent	Valid Percent	Cumulative Percent
Valid	Not Sure	11	20.8	22.4	22.4
	No	2	3.8	4.1	26.5
	Yes	36	67.9	73.5	100.0
	Total	49	92.5	100.0	
Missing	System	4	7.5		
Total		53	100.0		

54.7% were self motivated to get a vaccine

Motivation_to_get_vaccine

		Frequency	Percent	Valid Percent	Cumulative Percent
Valid	Family	16	30.2	32.7	32.7
	Job requirement	3	5.7	6.1	38.8
	My Care Giver	1	1.9	2.0	40.8
	Myself	29	54.7	59.2	100.0
	Total	49	92.5	100.0	
Missing	System	4	7.5		
Total		53	100.0		

Recommendation_to_Others

		Frequency	Percent	Valid Percent	Cumulative Percent
Valid	No	4	7.5	8.2	8.2
	Only if needed	15	28.3	30.6	38.8
	Yes	30	56.6	61.2	100.0
	Total	49	92.5	100.0	
Missing	System	4	7.5		
Total		53	100.0		

Booster_for_Children

		Frequency	Percent	Valid Percent	Cumulative Percent
Valid	No	16	30.2	32.7	32.7
	Natural Immunity	24	45.3	49.0	81.6
	Yes	9	17.0	18.4	100.0
	Total	49	92.5	100.0	
Missing	System	4	7.5		
Total		53	100.0		

54.7% recommended and endorsed compulsory vaccine policy among students

Vaccination_compulsion_students

		Frequency	Percent	Valid Percent	Cumulative Percent
Valid	No	5	9.4	10.2	10.2
	Ethical Approval	15	28.3	30.6	40.8
	Yes	29	54.7	59.2	100.0
	Total	49	92.5	100.0	
Missing	System	4	7.5		
Total		53	100.0		

41.5% thinks that vaccine is not suitable for pregnant

Vaccination_for_Pregnants

		Frequency	Percent	Valid Percent	Cumulative Percent
Valid	Not Sure	22	41.5	44.9	44.9
	No	15	28.3	30.6	75.5
	Yes	12	22.6	24.5	100.0
	Total	49	92.5	100.0	
Missing	System	4	7.5		
Total		53	100.0		

69.8% had no effect on sleep

Vaccination_Effect_on_Sleep

		Frequency	Percent	Valid Percent	Cumulative Percent
Valid	Just timely after few weeks it was normal	7	13.2	14.3	14.3
	No	37	69.8	75.5	89.8
	Yes	5	9.4	10.2	100.0
	Total	49	92.5	100.0	
Missing	System	4	7.5		
Total		53	100.0		

79.6% had no effect on Mental health

Vaccination_Effect_MH

		Frequency	Percent	Valid Percent	Cumulative Percent
Valid	Not Sure	5	9.4	10.2	10.2

	No	39	73.6	79.6	89.8
	Yes	5	9.4	10.2	100.0
	Total	49	92.5	100.0	
Missing	System	4	7.5		
Total		53	100.0		

66% favored compulsory vaccination policy

Compulsory_Vaccination_Views

		Frequency	Percent	Valid Percent	Cumulative Percent
Valid	It was a good decision	35	66.0	71.4	71.4
	It was hard for me to imply by all rules	8	15.1	16.3	87.8
	No it was bad decision as people should have freedom of choice	6	11.3	12.2	100.0
	Total	49	92.5	100.0	
Missing	System	4	7.5		
Total		53	100.0		
