# Supplementary material for: Attitude and Acceptance towards COVID-19 Booster Doses among Literacy Advantaged Population in Pakistan: A Cross-Sectional Study
Source: Vaccines (Basel). 2023 Jul 14;11(7):1238. doi: 10.3390/vaccines11071238 (PMC10383426; doi:10.3390/vaccines11071238)
Supplement: Supplementary file 1 [file vaccines-11-01238-s001.zip › STROBE Checklist.pdf]

STROBE Statement—Checklist of items that should be included in reports of ***cross-sectional studies***

**Title and abstract**	1	✔
	**Introduction**	
Background/rationale	2	✔
Objectives	3	✔
	**Methods**	
Study design	4	✔
Setting	5	✔
Participants	6	✔
Variables	7	✔
Data sources/ measurement	8	✔
Bias	9	✔
Study size	10	✔
Quantitative variables	11	✔
Statistical methods	12	✔
	**Results**	
Participants	13	✔
Descriptive data	14	✔
		✔
Outcome data	15	✔
Main results	16	✔
	**Discussion**	
Key results	18	✔
Limitations	19	✔
Interpretation	20	✔
	**Other information**	
Funding	21	✔
